# Supplementary material for: An Immunochromatographic Assay for the Rapid and Qualitative Detection of Mercury in Rice
Source: Biosensors (Basel). 2022 Aug 28;12(9):694. doi: 10.3390/bios12090694 (PMC9496535; doi:10.3390/bios12090694)
Supplement: Supplementary file 1 [file biosensors-12-00694-s001.zip › biosensors-1883199-supplementary.pdf]

Supporting information

An Immunochromatographic Assay for the Rapid and Qualitative Detection of Mercury in Rice

List of contents

1. The pretreatment of rice samples for ICP-MS.

2. Table S1. ICP-MS determination of mercury in real samples (n=4)

3. Figure S1. The standard curve of mercury for ICP-MS (y=192.468+1022.726x).

1. The pretreatment of rice samples for ICP-MS.

Each sample was weighed to 0.2 g in five copies, and three sets of blank controls were set up. 6ml of concentrated nitric acid was added to the samples and left to stand overnight. Microwave digestion was carried out the following day (at 190 °C for 20 min) and after completion of digestion, fixing the sample volume to 10 ml with 10% nitric acid and left to stand. Before the assay, a standard curve for mercury needs to be established. The column was cleaned and the parameters (RF power 1500 w, plasma gas flow rate 15 L min^{-1}, atomization temperature 2 °C, detection mode automatic) were set up before the sample was taken and the samples were injected one by one. The cps (counts per second) of the samples were obtained. The cps values were then substituted into the standard curve (y=192.468 + 1022.726x) equation to obtain the mercury content of the samples, which were diluted fifty times during the pre-treatment process.

Tables and artwork

Table S1. ICP-MS determination of mercury in real samples (n=4)

Figure S1. The standard curve of mercury for ICP-MS (y=192.468 + 1022.726x).

Table S1. ICP-MS determination of mercury in real samples (n=4).

Sample	Hg 202 (cps)	Real content (ng g^{-1})
1* rice	46297.99 ± 494.59	2287.5 ± 10.10
2* rice	10549.97 ± 54.47	507 ± 22.21
3* rice	1333.46 ± 39.68	58.5 ± 0.691
4* rice	290.3 ± 9.73	5.4 ± 0.23

Notes: +, positive; −, negative; ±, weak positive; cps, which is a ratio of the number of ions in the sample impinging on the detector per second / the number of ions in the internal standard impinging on the detector per second.

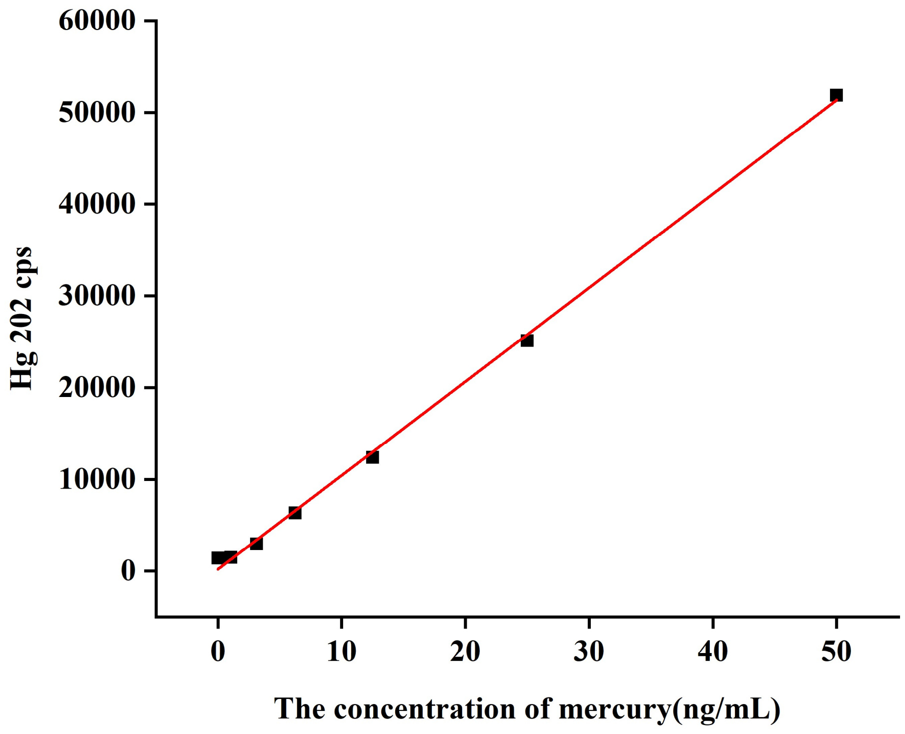

Figure S1. The standard curve of mercury for ICP-MS (y=192.468 + 1022.726x).
